# Efficacy of infiltrative local anesthesia and inferior alveolar nerve block in periapical surgery of lower premolars and molars: A preliminary report

**DOI:** 10.4317/jced.56278

**Published:** 2020-06-01

**Authors:** Isabel Menéndez-Nieto, Miguel Peñarrocha-Diago, Juan Cervera-Ballester, María Peñarrocha-Diago, David Peñarrocha-Oltra

**Affiliations:** 1 Master in Oral Surgery and Implant Dentistry, Stomatology Department, Faculty of Medicine and Dentistry, University of Valencia, Spain; 2 Chairman of Oral Surgery, Stomatology Department, Faculty of Medicine and Dentistry, University of Valencia, Spain; 3 Collaborating Professor of the Master in Oral Surgery and Implant Dentistry, Stomatology Department, Faculty of Medicine and Dentistry, University of Valencia, Spain; 4 Full Professor of Oral Surgery, Stomatology Department, Faculty of Medicine and Dentistry, University of Valencia, Spain; 5 Assistant Professor of Oral Surgery, Stomatology Department, Faculty of Medicine and Dentistry, University of Valencia, Spain

## Abstract

**Background:**

The aims of this study were: 1) compare the amount of anesthesia used with the anesthetic technique; 2) relate the quantity of anesthesia needed with the level of anxiety of the patient; 3) study the relationship between the anesthetic technique and the level of hemostasis; 4) correlate the amount of anesthesia with patient and tooth dependent variables.

**Material and Methods:**

A randomized controlled trial was designed with two parallel groups according to the anesthetic technique: infiltrative local anesthesia (infiltrative group) and inferior alveolar nerve block (block group). The following variables were collected: sex, age, smoking habits, plaque index, symptoms, signs, position of the tooth and amount of anesthesia. Before surgery, all patients were asked to assess their anxiety on a six-item questionnaire, the Amsterdam Preoperative Anxiety and Information Scale (APAIS). The analysis of the hemorrhage control of aluminum chloride was judged by the surgeon and two examiners independently and recorded it as: 0 (no hemorrhage control), 1 (slight but apparent intermittent bleeding persisted after application of the material), or 2 (complete hemorrhage control).

**Results:**

Twenty patients were included in this preliminary report. The amount of anesthesia used was lower in block group and in less anxious patients, although these results did not reach statistical significance. A relationship was found between the quantity of anesthesia used and a good hemostasis of the bony crypt before the application of the hemostatic agent (*p*<.05); and between elderly patients and a lower amount of anesthetic reinforcement (*p*<.05).

**Conclusions:**

Based on these preliminary results, we can conclude that no statistical significance difference was found between the amount of anesthesia used and the anesthetic technique or the anxiety. A relationship was found between hemostasis of the bony crypt and the quantity of anesthesia used; and between younger patients and a greater amount of anesthetic reinforcement.

** Key words:**Anesthesia, anxiety, endodontic surgery, hemostasis, hemostatic agents,periradicular surgery.

## Introduction

The generation of effective intra-operative anesthesia and hemostasis are critical pillars supporting the foundation of effective periapical surgery procedures (1). These achievements negate patient discomfort during the procedure and for a significant period (2) thereafter, while improving visual access in the surgical site minimizing surgical time, enhancing the surgical procedures (root-end resection, preparation and filling) (3-6), and reducing surgical blood loss, postoperative hemorrhage, and postoperative swelling (2,5). Anxious people are known to provide a higher score than non-anxious people in response to the same pain (7-9), therefore, the amount of anesthesia may be related to the level of anxiety of the patient.

The anesthetic techniques used in the lower premolar and molar region are infiltrative local anesthesia and inferior alveolar nerve block (IANB). In the literature, it was recommended to perform an IANB and once regional anesthesia has been achieved, a local infiltration over the flap to reduce bleeding in periapical surgery (10-12). To achieve hemostasis, it has been described that it is essential to use a local anesthetic solution containing an adrenergic vasoconstrictor (13,14); perform infiltrative anesthetic techniques to deposit the anesthesia-vasoconstrictor solution adjacent to the root apices (2); and infiltrate the anesthetic solution 5-10 minutes prior to any incisions (2,15).

Periapical surgery is performed when intracanal approaches are technically difficult or impractical, and affects the apical or lateral region surrounding a pulpless tooth (16). Therefore, the objective of the anesthetic technique will be anesthesia and vasoconstriction of the operative zone, not the pulp tissue. There is no article in the literature that relates the anesthetic technique used to the level of anesthesia and hemostasis of the surgical field in periapical surgery.

The aims of this study were: 1) compare the amount of anesthesia used with the anesthetic technique; 2) relate the quantity of anesthesia needed with the level of anxiety of the patient; 3) study the relationship between the anesthetic technique and the amount of anesthesia with the level of hemostasis; 4) correlate the amount of anesthesia with patient and tooth dependent variables.

## Material and Methods

-Study design

A randomized controlled trial was performed following the CONSORT guidelines for clinical trials (17), in the Oral Surgery Department (Faculty of Medicine and Dentistry, University of Valencia, Spain) from June 2015 to January 2018.

All patients had given their prior informed consent and the protocol was approved by the Ethical Committee of University of Valencia (H1481198441228), Spain.

Criteria for inclusion were: periapical lesions involving a single tooth in the lower premolar or molar region. Criteria for exclusion were: apico-marginal defects or teeth with periapical pathology associated with a vertical fracture.

Patients were randomly allocated into two parallel groups according to the anesthetic technique: infiltrative local anesthesia (infiltrative group) and inferior alveolar nerve block (block group). A block randomization scheme was generated by using the Web site Randomization.com (http://www.randomization.com) (seed: 153). The assignment was concealed from the surgeon until the time to perform the anesthetic technique by using an opaque envelope. The following variables were collected: sex, age, smoking habits (non-smokers, light smokers (≤10 cigarettes/day) or heavy smokers (>10 cigarettes/day)), plaque index (18), symptoms (asymptomatic, pain, inflammation or pain and inflammation), signs (no alterations, swelling or fistula), position (first premolar, second premolar, first molar, second molar) and amount of anesthesia (measured in milliliters).

**Figure 1:**
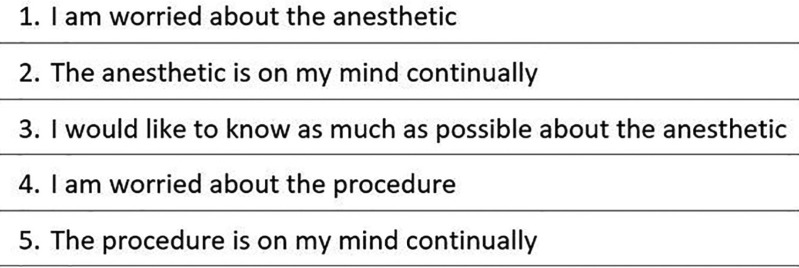
The Amsterdan Preoperative Anxiety and Information Scale (APAIS).

Before surgery, all patients were asked by a blinded interviewer to assess their anxiety on a six-item questionnaire, the Amsterdam Preoperative Anxiety and Information Scale (APAIS) developed in 1996 (19). The measure of agreement with these statements (Fig. 1) was graded on a five-point Likert scale from 1 (not at all) to 5 (extremely). Higher scores indicate higher levels of anxiety and desire for information.

The surgeon (M.P.D) and two independent blinded examiners (I.M.N; J.C.B) judged the bleeding before and after the application of the hemostatic agent. For hemostasis evaluation the classification proposed by Peñarrocha-Diago *et al.* (20) was used.

- 0: No hemorrhage control, continuous or intermittent bleeding that compromised root-end filling procedures.

- 1: Slight but apparent intermittent bleeding that allowed root-end filling procedures.

- 2: Complete hemorrhage control that allowed root-end filling procedures.

During the surgical procedure, the surgeon recorded the value and the bony crypts were photographed (Canon EOS 70D, Canon Macro Ring Lite MR-14EX, Canon EF 100mm f/2.8 Macro USM; Tokyo, Japan). A PDF (Adobe Acrobat Reader DC, Adobe Systems Inc., San Jose, CA, USA) document was given to each observer with the clinical photographs for its viewing on a 21.5-inch monitor (iMac, Apple, CA, USA) with a screen resolution of 4096 x 2304 pixels.

-Surgical Technique 

All interventions were performed by the same surgeon. In infiltrative group, an infiltrative local anesthesia of 1,8mL was accomplished with 4% articaine and 1:100.000 epinephrine (Inibsa®, Lliça de Vall, Barcelona, Spain). In block group, an inferior alveolar nerve block of 1,8 mL with the same anesthetic was administered. If the patient had discomfort during the surgery, additional infiltrations of 0.9mL were performed, using the same anesthetic technique in each group: infiltrative in infiltrative group and inferior alveolar nerve block in block group. After 10 minutes, a mucoperiosteal flap with submarginal incision design was raised. After ostectomy the roots were resected and the tissue around the apex was removed. the root-end cavities were prepared with ultrasonic retro-tips (Piezon® Master 400 EMS, Electro Medical Systems S.A, Switzerland) to a 3 mm depth.

Hemostasis was performed with aluminum chloride (Expasyl™, Produits Dentaires Pierre Rolland, Merignac, France) applied into the bone defect for 2 minutes. The retrograde cavities were filled with mineral trioxide aggregate (MTA; Dentsply® Tulsa Dental, Tulsa, OK, USA) and their quality was evaluated with a rigid endoscope (Möller-Wedel®, Munich, Germany). After washing the cavity with sterile saline and a curette, the superficial bone layer was removed with rotary instruments. Primary wound closure was accomplished with 6/0 multiple interrupted sutures (Polinyl, Sweden & Martina, Carrare, Italy) suture.

Preoperative antibiotic prophylaxis was administered with 2 g of amoxicillin-clavulanic acid 1 hour before surgery. The following medication was prescribed: 600mg ibuprofen as needed and 0.12% chlorhexidine rinses 2 times a day for 7 days. Suture were removed after 7 days.

-Statistical analysis

The study was blinded for the patients and the biostatistician with experience in dentistry: the patients were not informed of the anesthetic technique performed and the statistician received a database divided into groups “I” and “II”, without specifying the assignment groups. The statistical analysis was performed with the Statistical Package for Social Science (SPSS, version 8.0 for Windows; SPSS Inc, Chicago, IL, USA). A *p*-value <0.05 was considered significant. A descriptive analysis of all variables was made: mean, standard deviation, minimum and maximum, and absolute and relative frequencies for categorical parameters. The Chi2 test and the Fisher exact test were used to determine the degree of association between two variables of categorical type. The Mann-Whitney U-test was used to compare the amount of additional anesthesia between the two groups and its relationship with patient and tooth dependent variables. Spearman correlation test was used to estimate the relationship between the level of anxiety and the amount of anesthesia used. Cohen’s kappa statistic was applied to assess interobserver agreement between the three examiners.

## Results

**Figure 2:**
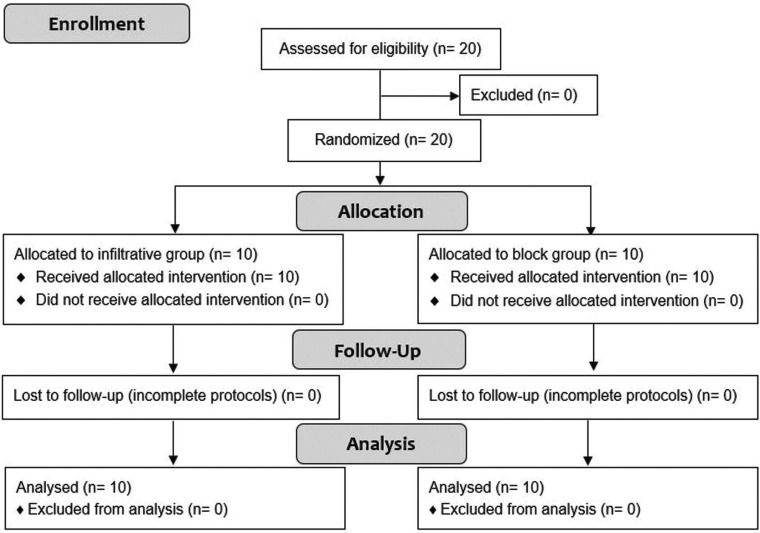
Consort flow diagram.

No patient was excluded, and all 20 patients were included in this preliminary report and assigned to two groups (10 in each group) depending on the anesthetic technique used (Fig. 2). The patient sample consisted of 14 female patients and 6 male patients, with a mean age of 48.6 years (SD 16.7) ( Table 1) and a lower premolar or molar affected by a periradicular lesion.

**Table 1:**
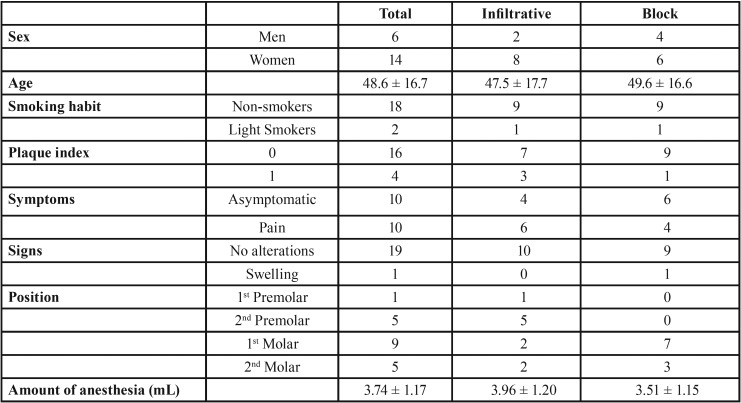
Descriptive statistics of patients included in the study.

- Infiltrative group: one first premolar, five second premolars, two first molars and two second molars.

- Block group: seven first molars and three second molars.

No statistically significant differences were found between the amount of additional anesthesia used and the anesthetic technique (*p*>0.05). However, in 50% of the patients of the infiltrative group, 1.8mL of anesthesia was not enough compared to 30% of the patients of the block group. The mean amount of anesthesia used was 3.96mL (SD 1.20) and 3.51mL (SD 1.15), in the infiltrative group and in the block group, respectively.

Both groups were homogeneous in the level of previous anxiety (*p*>0.05). Total APAIS score of the cases ranged between 6 and 18, and the mean was 11.50 (SD 3.26) ( Table 2). No statistically significant differences were found between the amount of additional anesthesia used and the level of anxiety of the patient (*p*>0.05). However, when the concern for anesthesia was “not at all” or “slightly”, 50% of the patients did not need more than 1.8mL of anesthetic reinforcement; and when the concern was “moderately” or “very”, 50% of the patients needed more than 2.7mL of anesthetic reinforcement.

**Table 2:**
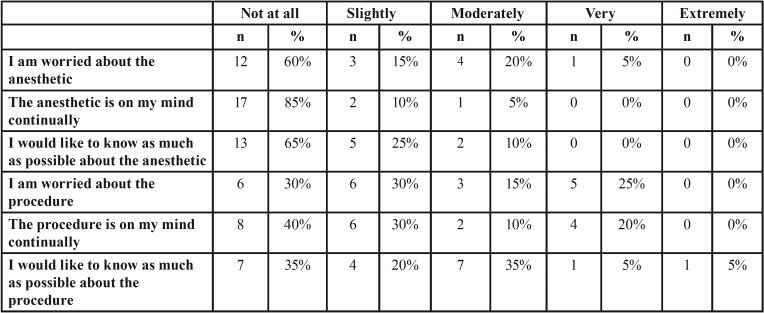
APAIS scale distribution.

Regarding hemostasis, aluminum chloride was used in 85% (n=17) of the patients; 70% in the infiltrative group and 100% in the block group (*p*>0.05). In the three patients in whom it was not necessary to place the hemostatic agent (infiltrative group), anesthesia was reinforced with 2.7 mL; a statistically significant difference was found between the amount of anesthesia used and the hemostasis of the bony crypt before the application of the hemostatic agent (*p*<0.05). The impact of the amount of anesthesia is correlated to the use of infiltrative anesthesia techniques, however, due to the low sample size, no statistically significant differences were found (*p*> 0.05) ( Table 3).

**Table 3:**
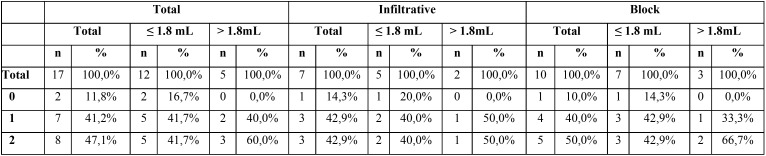
Hemostatic efficacy of aluminum chloride according to the anesthetic technique and the additional anesthesia used.

Regarding the hemostatic efficacy after using aluminum chloride (n=17): a complete hemorrhage control was achieved in 8 patients (47%) and a slight but apparent intermittent bleeding was found in 7 patients (41.2%) and no hemorrhage control in 2 patients (11.8%) ( Table 3). In those cases, in which control of bleeding was not achieved, a gauze impregnated in epinephrine (B-Braun, 1 mg/mL; Rubí, Barcelona, Spain) was used to avoid compromising the quality of the root-end filling procedures. A complete hemorrhage control was found in 41.7% of the patients in whom less than 1.8 mL of anesthetic reinforcement was needed and in 60% of the patients when the reinforcement was greater than 1.8 mL (*p*>0.05) ( Table 3).

The variables collected (sex, age, smoking habits, plaque index, symptoms, signs and position) were related to the amount of anesthesia used. Only age significantly affected the amount of reinforcements needed, it was observed that a lower number of milliliters of anesthesia was used at an older age (*p*<0.05).

The calculated kappa values of the pairwise comparisons (three examiners) were 0.802, 0.702 and 0.901, an average value of 0.802. These values indicated a good concordance between the three observers.

## Discussion

The aim of this study was to compare the amount of anesthesia and the anesthetic technique (infiltrative or inferior alveolar nerve block) used with patient anxiety and hemostasis of the bony crypt, during periapical surgery of premolars and lower molars; and its relationship with patient and tooth dependent variables.

The preliminary results of the present prospective study, despite the limitations due to the small number of patients, indicated that the amount of anesthesia used was lower in block group, as well as, in less anxious patients, although these results did not reach statistical significance. In addition, the use of a greater amount of anesthesia was associated with young patients and with better hemostasis of the bone crypt.

Currently, there are no studies in the literature that analyze the anesthetic efficacy of infiltrative anesthesia and inferior alveolar nerve block in pulpless posterior mandibular tooth, as occurs in periapical surgery.

Klages *et al.* (21) conducted a study to investigate the relationship between anxiety and pain experienced during dental treatment. They concluded that highly anxious patients reported more pain during interventions, compared to low fearful patients. The present study found that anxious patients needed a greater amount of anesthesia, however this result was not statistically significant.

Menéndez-Nieto *et al.* (22) reported a complete hemostasis of 72.5% when using aluminum chloride as hemostatic agent; and Peñarrocha-Diago y *cols.* (23) described that complete control of bleeding occurred in 80% of patients and in 20% a slight but apparent intermittent bleeding when they used the same agent. In this study, it was found that in 47% of the patients the control of the hemorrhage was complete and in 42.1% the bleeding was intermittent but apparent. This may be due to the fact that we avoid placing a large amount of the agent in premolars and lower molars, since access for its removal of the bone crypt is complicated. If the aluminum chloride is not removed correctly, it produces a foreign body reaction and a delayed bone formation (24-26).

The relationship between dental anxiety and patient depend variables has been more studied. Enkling *et al*. (27), Hakeberg *et al.* (28) and Thomson *et al.* (29) showed that younger people was more afraid of dental treatment than older people. The highest level of fear was found in the groups of 20-30, 20-39 and 35-44 years old, respectively. The same authors found that women are more anxious than men (27-29). This result must be evaluated critically, though, as women may simply be more willing to admit and express their feelings of fear in conjunction with dental treatment (27). The present study found that younger patients needed a greater amount of anesthesia and this may be related to the level of anxiety. However, we did not find a relationship between sex and anxiety or amount of anesthesia used.

Inferior alveolar nerve block supplemented with infiltrative anesthesia is usually the technique of choice for periapical surgery of lower premolars and molars (10-12). However, IANB may be more difficult technically to perform and it has additional disadvantages, including the potential for causing nerve damage and the failure to counter any accessory nerve supply (30).

Despite randomization, no premolar was assigned to the block group, therefore the outcome should be interpreted with caution. This fact was analyzed in terms of anxiety and it was concluded that the position of the tooth does not seem to be related to anxiety in the patient, so no special control measures were taken on this point in the analysis. Further studies with a randomized controlled design and a large sample are needed to confirm our results.

## Conclusions

Based on these preliminary results, we can conclude that no statistical significance difference was found between the amount of anesthesia used and the anesthetic technique or the anxiety. A relationship was found between hemostasis of the bony crypt and the quantity of anesthesia used; and between younger patients and a greater amount of anesthetic reinforcement was needed.

## References

[1] Hargreaves KM, Khan A (2005). Surgical preparation: anesthesia & hemostasis.

[2] Gutmann JL (1993). Parameters of achieving quality anesthesia and hemostasis in surgical endodontics. Anesth Pain Control Dent.

[3] Selden HS (1970). Bone wax as an effective hemostat in periapical surgery. Oral Surg Oral Med Oral Pathol.

[4] Kim S, Rethnam S (1997). Hemostasis in endodontic microsurgery. Dent Clin North Am.

[5] Witherspoon DE, Gutmann JL (1996). Haemostasis in periradicular surgery. Int Endod J.

[6] Cho YW, Kim E (2013). Is stopping of anticoagulant therapy really required in a minor dental surgery? - How about in an endodontic microsurgery?. Restor Dent Endod.

[7] Cho SY, Kim E, Park SH, Roh BD, Lee CY, Lee SH (2017). Effect of Topical Anesthesia on Pain from Needle Insertion and Injection and Its Relationship with Anxiety in Patients Awaiting Apical Surgery: A Randomized Double-blind Clinical Trial. J Endod.

[8] Means-Christensen AJ, Roy-Byrne PP, Sherbourne CD, Craske MG, Stein MB (2088). Relationships among pain, anxiety, and depression in primary care. Depress Anxiety.

[9] Kent G (1984). Anxiety, pain and type of dental procedure. Behav Res Ther.

[10] Niemczyk SP (2010). Essentials of Endodontic M i c ro s u r g e r y. Dent Clin NA.

[11] Wang N, Knight K, Dao T, Friedman S (2004). Treatment outcome in endodontics-The Toronto Study. Phases I and II: apical surgery. J Endod.

[12] Carrotte P (2005). Surgical endodontics. Br Dent J.

[13] Jastak JT, Yagiela JA (1983). Vasoconstrictors and Local Anesthesia: A Review and Rationale for Use. J Am Dent Assoc.

[14] Molven O, Halse A, Grung B (1991). Surgical management of endodontic failures: indications and treatment results. Int Dent J.

[15] Gutmann JL, Frazier Jr LW, Baron B (1996). Plasma catecholamine and haemodynamic responses to surgical endodontic anaesthetic protocols. Int Endod J.

[16] Löst C (2006). Quality guidelines for endodontic treatment: Consensus report of the European Society of Endodontology. Int Endod J.

[17] Schulz KF, Altman DG, Moher D, CONSORT Group (2010). CONSORT 2010 statement: updated guidelines for reporting parallel group randomised trials. BMJ.

[18] Silness J, Loe H (1964). Periodontal disease in pregnancy. II. correlation between oral hygiene and periodontal condtion. Acta Odontol Scand.

[19] Moerman N, van Dam FS, Muller MJ, Oosting H (1996). The Amsterdam Preoperative Anxiety and Information Scale (APAIS). Anesth Analg.

[20] Peñarrocha-Diago M, Menéndez-Nieto I, Cervera-Ballester J, Maestre-Ferrín L, Blaya-Tárraga JA, Peñarrocha-Oltra D (2018). Influence of Hemostatic Agents in the Prognosis of Periapical Surgery: A Randomized Study of Epinephrine versus Aluminum Chloride. J Endod.

[21] Klages U, Ulusoy Ö, Kianifard S, Wehrbein H (2004). Dental trait anxiety and pain sensitivity as predictors of expected and experienced pain in stressful dental procedures. Eur J Oral Sci.

[22] Menéndez-Nieto I, Cervera-Ballester J, Maestre-Ferrín L, Blaya-Tárraga JA, Peñarrocha-Oltra D, Peñarrocha-Diago M (2016). Hemostatic Agents in Periapical Surgery: A Randomized Study of Gauze Impregnated in Epinephrine versus Aluminum Chloride. J Endod.

[23] Peñarrocha-Oltra D, Menéndez-Nieto I, Cervera-Ballester J, Maestre-Ferrín L, Peñarrocha-Diago MA (2019). Aluminum Chloride versus Electrocauterization in Periapical Surgery : A Randomized Controlled Trial. J Endod.

[24] von Arx T, Jensen SS, Hänni S, Schenk RK (2006). Haemostatic agents used in periradicular surgery: An experimental study of their efficacy and tissue reactions. Int Endod J.

[25] Jensen SS, Yazdi PM, Hjørting-Hansen E, Bosshardt DD, von Arx T (2010). Haemostatic effect and tissue reactions of methods and agents used for haemorrhage control in apical surgery. Int Endod J.

[26] Mena-Álvarez J, Quispe-López N, Zubizarreta-Macho Á, Rico-Romano C, Rodero-Villanueva R, Fernández-Aceñero MJ (2019). Histological analysis of different local haemostatic agents used for periapical surgery: An experimental study with Sprague-Dawley rats. Aust Endod J.

[27] Enkling N, Marwinski G, Jöhren P (2006). Dental anxiety in a representative sample of residents of a large German city. Clin Oral Investig.

[28] Hakeberg M, Berggren U, Carlsson SG (1992). Prevalence of dental anxiety in an adult population in a major urban area in Sweden. Community Dent Oral Epidemiol.

[29] Thomson WM, Stewart JF, Carter KD, Spencer AJ (1996). Dental anxiety among Australians. Int Dent J.

[30] Meechan JG (2011). The use of the mandibular infiltration anesthetic technique in adults. J Am Dent Assoc.

